# Utility of artificial intelligence in predicting clinical outcomes of angiographic procedures: a comprehensive scoping review

**DOI:** 10.1186/s44348-026-00064-x

**Published:** 2026-08-20

**Authors:** Shreeyash Shashank Tulpule, Sammita Jagdish Jadhav

**Affiliations:** Symbiosis Institute of Health Sciences, Symbiosis International (Deemed University), Pune, India

**Keywords:** Artificial intelligence, Coronary Angiography, Risk Stratification, Clinical Decision making, Deep learning, Review, Diagnostic Imaging

## Abstract

Artificial intelligence (AI) is emerging as a transformative tool in cardiovascular imaging, particularly in coronary angiography. With the growing interest in precision medicine, AI offers the potential to enhance clinical decision-making by improving diagnostic accuracy, predicting outcomes, and guiding interventions. This scoping review aims to examine the current landscape of AI applications in predicting clinical outcomes related to angiographic procedures, including diagnosis, risk stratification, and postprocedural monitoring. A total of 59 relevant studies published between 2015 and 2025 were included in this review. The selection was based on predefined inclusion criteria focused on AI-based models applied in coronary artery disease diagnosis, outcome prediction and restenosis detection. The studies were reviewed for the AI techniques used, clinical endpoints targeted, model performance, validation strategies, and limitations. Four primary themes were identified: (1) prediction of adverse cardiovascular events such as myocardial infarction and mortality; (2) detection of hemodynamically significant stenosis; (3) prediction of in-stent restenosis; and (4) identification of coronary arteries. Machine learning algorithms, especially random forests, support vector machines, and convolutional neural networks, have been commonly used. Many models have demonstrated superior performance compared to traditional statistical methods. However, limitations such as small sample sizes, lack of external validation, retrospective designs, and concerns about data privacy were frequently observed in these studies. AI demonstrates promising capabilities in outcome prediction within coronary angiography. Despite current limitations, its integration into clinical practice is feasible with prospective, multicentric studies and standardized validation frameworks. Ethical considerations and regulatory oversight will be crucial in ensuring safe and effective implementation.

## Background

Application of artificial intelligence (AI) is transforming medicine on three levels: for clinicians, through quick and precise image interpretation; for health systems, through enhanced workflow and the possibility of lowering medical errors; and for patients, by empowering them to process their own data to improve health [1]. Since its introduction by Dr. Mason Sones in 1958, coronary angiography has continued to be the gold standard for assessing coronary artery disease (CAD). It provides information on the extent of collateral circulation as well as the location and severity of the CAD [2]. Cardiac Catheterization labs churn out huge amount of data in the form of high frame cine-images, pressure recordings or real-time hemodynamic data, which can be beneficial as this data can be useful in improving diagnosis and treatment, prediction of disease and outcomes along with pharmacovigilance and safety of the patient [3]. In addition, the operator and patient safety can also be enhanced by use of AI models in the coronary angiography workflow. AI included decision-support systems, present a comprehensive picture of all diagnostic parameters, helps lower procedural risks and avoid unnecessary complications caused by non-essential interventions of the patient [4].

AI methods are seen to outperform conventional statistical models and predictive scores such as SYNTAX score, thrombolysis in myocardial infarction (TIMI) score and GRACE (Global Registry of Acute Coronary Events) scores, in terms of prediction [5, 6] The procedure of invasive angiography has certain complications like bleeding, hematoma, acute renal failure, contrast-induced nephropathy along with false aneurysms [7]. Compared to clinical risk factors for major adverse cardiac events (MACEs) in patients, deep learning (DL)-based diagnosis of obstructive CAD showed a higher predictive value [8]. The primary objective of this review is to examine existing literature on the applications of AI for predicting clinical outcomes in patients undergoing angiographic procedures. Specifically, the review aims to categorize the types of AI algorithms used; identify the nature of data inputs and predicted outcomes; evaluate model performance and validation strategies; and explore challenges and opportunities in translating these tools into routine clinical practice.

## Methods

This scoping review adheres to the methodological framework developed by Arksey and O’Malley (2005), further enhanced by the PRISMA-ScR (Preferred Reporting Items for Systematic Reviews and Meta-Analyses Extension for Scoping Reviews) guidelines [9]. This structured approach facilitates a comprehensive mapping of existing literature, identifies key concepts, and highlights gaps in knowledge related to the application of AI in predicting clinical outcomes following angiographic procedures. The Critical Appraisal Skills Programme (CASP) quality checklist was used to evaluate and 59 were included with a minimum score of 16, in which 36 articles scored between 16–20, and 21 articles scored above 20 up to score 24.

### Search strategy

**Fig. 1:**
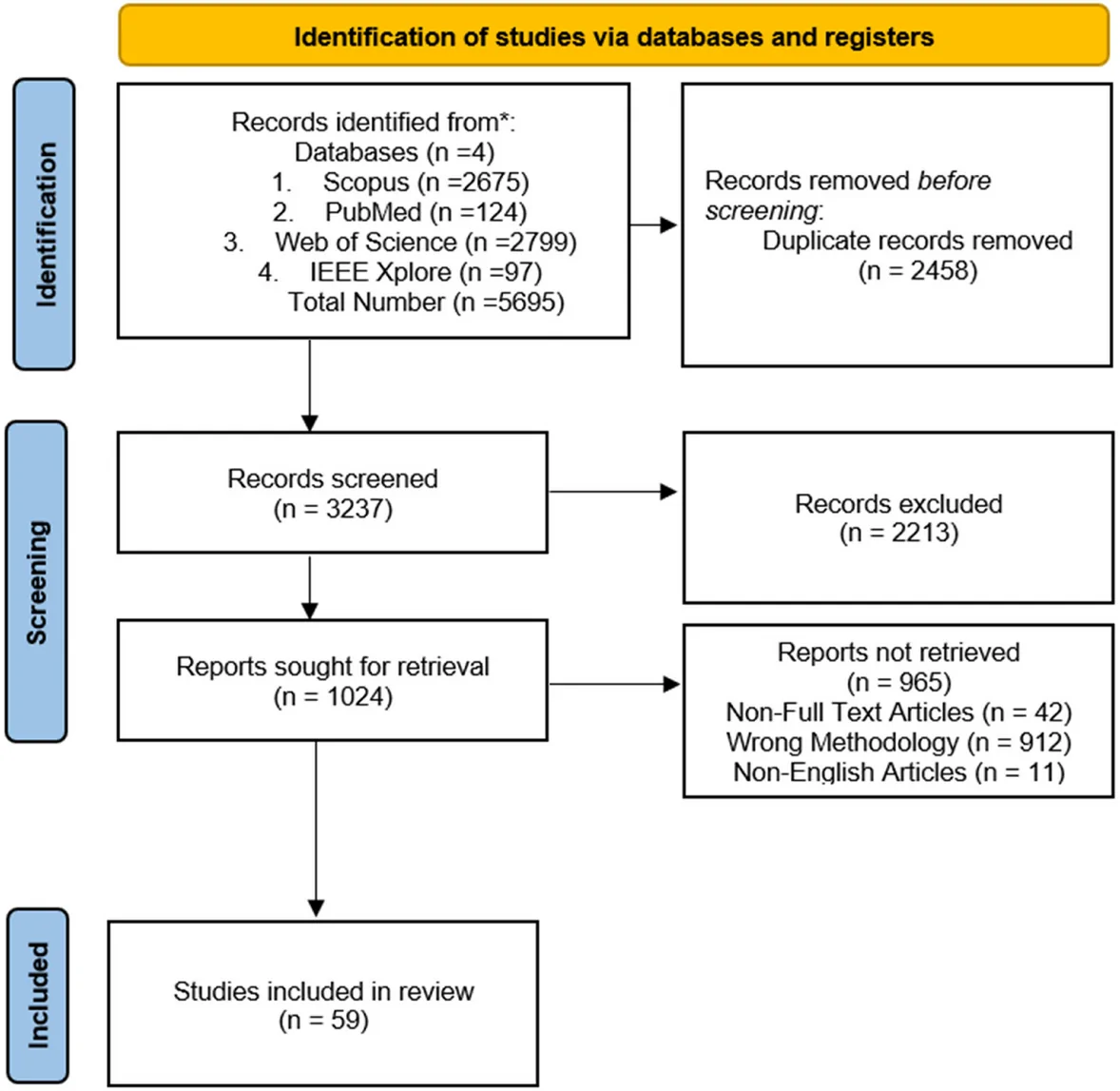
PRISMA (Preferre Reporting Items for Systematic Reviews and Meta-Analyses) flowchart

A systematic and exhaustive literature search was undertaken to identify relevant studies exploring the application of AI in predicting clinical outcomes following angiographic procedures. The search was conducted across four major electronic databases (PubMed, Scopus, Web of Science, and IEEE Xplore) to ensure comprehensive coverage of both biomedical and engineering literature. The search was restricted to peer-reviewed journal articles published between January 2015 and March 2025, thereby capturing a decade of advancements in AI methodologies and their integration into cardiovascular imaging and diagnostics as shown in Fig. 1. To maximize retrieval of pertinent studies, a combination of free-text keywords and MeSH (Medical Subject Headings) was employed. Key terms included “artificial intelligence,” and “angiography.”.

### Inclusion and exclusion criteria

Studies were included if they met the following criteria: (1) studies which employed AI or machine learning (ML) models to predict clinical outcomes of angiographic procedures; (2) studies involving human subjects undergoing angiography procedure; and (3) reported measurable performance metrics such as accuracy, sensitivity, specificity, or area under the curve (AUC). Non-English language studies conference abstracts without full texts were excluded.

### Data extraction

Data were extracted from eligible studies using the standard reporting form. Key parameters recorded included author and year of publication, study location, type of AI/ML algorithm used (e.g., convolutional neural network (CNN), support vector machine [SVM], random forest), imaging modality (e.g., coronary computed angiography (CCTA), intravascular ultrasound, conventional angiography), dataset characteristics (e.g., sample size, training/test split), predicted clinical outcomes (e.g., MACE, restenosis, mortality), and whether they incorporated real-time or retrospective analysis as shown in Table 1. Two researchers independently evaluated each article and made a decision on include or not and Cohen Ks was calculated. The Cohens K for this study was 0.78.

**Table 1:** Summary of AI applications in angiographic outcome prediction

Outcome type	Algorithm	Validation strategy	Parameter	Key finding
Adverse events (MI, AKI, mortality, no reflow)	RF, XGBoost, ANN, LASSO, logistic regression, CNN, NB	Internal split, cross-validation	Age, sex, comorbidities, lab values (CRP, creatinine, troponin), vitals, imaging	AUCs, 0.775–0.910 ANN outperformed linear models in radiation dose prediction
CAD detection (stenosis, occlusion, bridge)	CNN, XGBoost, RF, SVM, KNN, YOLO, U-Net, ConvMixer	Internal split, bootstrap, Dice/ASD	Coronary angiography, demographic data, plaque morphology	Dice scores up to 0.917 Multimodal fusion improved accuracy
In-stent restenosis	LASSO logistic, RF, XGBoost	Internal split only	Stent dimensions, lesion morphology, DAPT adherence, lipid profiles	Tree-based models handled class imbalance well Key predictors were stent-related
Readmission (CSFP)	Multivariate regression	Bootstrap internal validation	Age, sex, smoking, drinking, SBP, lipid profile, diabetes, blood counts	AUC, 0.875 Lacked external validation Psychosocial factors not included
Intramyocardial hemorrhage	Nomogram (univariate/bivariate regression)	Train/test split	SYNTAX score, myocardial enzymes, NT-proBNP	AUC, 0.893–0.910 No MACE follow-up Single-center data
Stenosis quantification (CT/QCA)	AI-CSQ, U-Net	Per-vessel and per-patient ROC	QCA, image quality metrics, calcium burden	AUCs, 0.88–0.93 Performance affected by image quality and plaque burden
Radiation dose prediction (CATHLAB)	ANN	Internal split	Dose-area product, procedural metadata	ANN achieved *R* = 0.95 Outperformed linear regression
AKI prediction in CKD patients	NN, NB, KNN, LR, RF, XGBoost	Temporal validation (single year)	Creatinine, BUN, demographic data, procedural details	AUCs, 0.648–0.733 Limited generalizability due to small sample
Mortality prediction from chest x-ray	DL-CXR	Internal and external validation	Chest x-ray, age, CAD status, comorbidities	AUC dropped from 0.79 to 0.71 on external data Interpretability concerns
In-stent restenosis risk score	LASSO-logistic	Registry-based internal split	Angiography images, procedural history	No external validation Performance in ACS patients remains untested

*ACS* acute coronary syndrome, *AKI* Acute Kidney Injury, *AI-CSQ* artificial intelligence-informed coronary stenosis quantification, *ANN* Advanced Neural Network, *ASD* Average Surface Distance, *AUC* Area under curve, *BUN* blood urea nitrogen, *CAD* Coronary Artery Disease, *CATHLAB* cardiac catheterization laboratory, *CKD* chronic kidney disease, *CNN* convolution neural network, *CRP* C-reactive protein, *CSFP* Coronary Slow Flow Phenomena, *CT* computed tomography, *DAPT* Dual Anti-Platelet Therapy, *DL-CXR* deep learning chest x-ray, *KNN* K-Nearest Neighbors, *LASSO* least absolute shrinkage and selection operator, *LR* Logistic Regression, *MACE* Major Adverse Cardiac Event, *MI* Myocardial Infarction, *NB* Naïve Bayes, *NN* Neural Network, *NT-pro BNP* N-terminal pro-brain natriuretic peptide, *QCA* quantitative coronary angiography, *RF* random forest, *ROC* receiver operating characteristic, *SBP* systolic blood pressure, *SVM* support vector machine, *XGBoost* Extreme Gradient Boosting

### Data analysis and thematic synthesis

A thematic synthesis approach was employed to analyze and categorize the extracted data. Studies were grouped based on outcome types, model types, imaging modalities, and integration into clinical practice. Common methodological themes, gaps in external validation, and barriers to clinical translation were identified through qualitative synthesis.

### Search results

An extensive search from four databases PubMed, Web of Science, and Scopus along with IEEE Xplore yielded 5,695 articles which were screened in Rayyan software. A total of 2485 duplicate articles were removed, and 2,213 articles were excluded from the study, revealing 1024 articles for full review. A total of 965 articles were excluded, and 59 articles were included in the final review. Grey literature was identified using Google Scholar.

## Results

### Study selection and characteristics

A total of 59 articles were included in this scoping review, each investigating the application of artificial intelligence (AI) in clinical settings. The studies on this topic present a notable increase in publications in recent years, reflecting the growing interest in AI-driven healthcare solutions, out of which 58 included studies are primary along with one case study [10]. The studies encompassed a wide range of cardiovascular conditions, including but not limited to CAD, heart failure, arrhythmias, and valvular heart disease. The AI/ML applications varied from diagnostic support and risk stratification to outcome prediction and image analysis [11, 12]. This diversity underscores the broad applicability and growing interest in leveraging AI/ML to address complex challenges in cardiovascular care.

The studies included show a growing trend from 2022 to 2024, with 2024 being the year with maximum number of publications. 9 studies have been published in the first half of 2025, signifying the growing interest in the topic [13–15]. All 59 studies, used different forms of AI used to predict outcomes of angiography procedure. Majority of the studies analyzed were primary studies with patient data used for training and validation of the model.

### Identified primary themes

Four primary themes were identified: (1) prediction of adverse cardiovascular events such as myocardial infarction and mortality; (2) detection of hemodynamically significant stenosis; (3) prediction of in-stent restenosis; and (4) identification of coronary arteries.

#### Predicting adverse events

Thirty—Four studies (60%) predicted various adverse events after angiography. Current investigations into peri-procedural risk increasingly focus on forecasting acute complications, major bleeding episodes, arrhythmic events, or contrast-related nephropathy, during the immediate postoperative or in-hospital period. These studies assemble expansive feature sets that blend demographic variables (age, sex, body-mass index, ethnicity, smoking status) as seen in Table 2 with continuously sampled physiologic data (sequential heart-rate and arterial-pressure readings, oxygen-saturation trends) and routinely obtained biochemical markers (serum creatinine, hemoglobin, high-sensitivity troponin, C-reactive protein) [16–18]. Following comprehensive data conditioning, including outlier moderation, multiple imputation of missing values, temporal summarization of waveform signals, and standardization, the inputs are analyzed with ensemble techniques, most commonly random-forest or gradient-boosting models. Such algorithms are preferred because they manage heterogeneous inputs, capture complex non-linear relationships, and provide interpretable feature-importance profiles suitable for clinical scrutiny. The overarching objective articulated across the studies was anticipatory care. Investigators envisage their models as continuously operating surveillance components embedded in electronic health-record (EHR) systems [19, 20]. By refreshing risk calculations whenever new vital-sign or laboratory information becomes available, the models can issue graded, real-time alerts that prompt clinicians to intervene before overt physiologic decline occurs. In harnessing detailed demographic information alongside dynamic physiologic and laboratory data, these ensemble approaches aim to shift peri-operative management from a reactive stance to a preventive one, thereby improving patient safety and optimizing resource utilization [21, 22].

**Table 2:** Summary of data sources and demographic characteristics of included studies

Characteristics	Summary
Data source	Predominantly retrospective hospital records and angiographic image datasets Only three studies used multicenter registries or public repositories
Geographic distribution	China (48%), USA (9%), Turkey (7%), Italy (5%), Germany (3%), Canada (3%), Iran, Kazakhstan, and others
Sample size	Range, 80 to 12,000 patients; median, ≈1,050 Small cohorts (< 500 patients) in 40% of studies
Age reporting	Reported in 90% of studies Mean age typically between 55–68 years
Sex distribution	Male predominance in 85% of studies Female representation often < 30%
Ethnicity and socioeconomic status	Ethnicity reported in < 10% of studies Socioeconomic status and education levels largely absent

#### Detection of hemodynamically significant stenosis

The second-largest group of studies was devoted to the non-invasive detection of hemodynamically significant coronary artery disease (CAD) with 18 studies (32%) predicted CAD as an outcome of angiography in patients who were suspected of coronary artery disease and underwent coronary angiography. These investigations center on high-resolution imaging, principally coronary computed tomography angiography (CCTA), supplemented in several instances by invasive coronary angiography or intravascular ultrasound frames exported for post-hoc analysis [23, 24]. Researchers preprocess the volumetric data through automated centerline extraction, isotropic resampling, and intensity normalization before feeding them into convolution neural-network CNN backbones such as U-Net, ResNet-50, or DenseNet-121 [25, 26]. The networks are trained to perform either voxel-level plaque segmentation or whole-vessel obstruction classification, with ground-truth labels derived from expert cardiologist readings, fractional-flow-reserve measurements, or quantitative coronary angiography (QCA). To move beyond purely anatomical cues, approximately one-third of the articles fuse the image-derived feature tensors with tabular clinical covariates (age, sex, systolic blood pressure, lipid profiles, glycated hemoglobin, and smoking exposure) using late-fusion strategies (concatenating embeddings just before the classification head) or dual-stream architectures in which parallel subnetworks process imaging and clinical data independently before joint decision-making [27, 28]. Authors argue that this multimodal integration captures both macroscopic plaque morphology and the systemic milieu that drives atherosclerosis, yielding a richer representation of disease burden than either modality can offer in isolation. The narrative thread across the studies emphasizes diagnostic streamlining. Investigators position their algorithms as “gatekeepers” capable of stratifying patients after an initial noninvasive scan but before invasive catheterization. In proof-of-concept simulations, the models theoretically avert a substantial fraction of non-therapeutic angiograms while maintaining high sensitivity for clinically actionable stenoses. Several studies further explore workflow implementation: automatically generated heatmaps pinpointing culprit plaques are overlaid on CCTA slices, and calibrated probability scores are exported to the radiology report or cardiac multidisciplinary-team dashboard. Collectively, this literature envisions an imaging-augmented, data-fused triage pathway that could reduce procedural risk, shorten diagnostic timelines, and allocate catheter-lab resources to the patients most likely to benefit from invasive intervention [29, 30].

#### Predicting in-stent restenosis

The in-stent restenosis literature represents the smallest yet most technically focused theme, with 4 (7%) predicting in-stent restenosis in follow-up angiographies in patients who had previously undergone percutaneous coronary interventions (PCIs). Investigators begin by extracting detailed procedural descriptors (stent length, normal diameter, lesion-segment morphology, and residual stenosis percentage) directly from the catheterization report or QCA software [31, 32]. These static anatomical metrics are then augmented with longitudinal follow-up data, most notably adherence to dual-antiplatelet therapy, interval lipid-profile trends, and, where available, pharmacogenomic information that affects clopidogrel metabolism. The resulting dataset is heavily imbalanced, as restenosis events are relatively infrequent compared with patent stents. To model this sparsity, researchers overwhelmingly favor tree-based algorithms (gradient-boosting machines and random forests) because they natively support class-weighting, handle mixed numeric-categorical inputs, and retain interpretability through variable-importance plots. Several studies apply synthetic-minority over-sampling or focal-loss functions to further mitigate imbalance. Feature-importance analyses consistently elevate stent dimensions and DAPT (Dual Anti-Platelet Therapy) adherence above traditional demographic variables, underscoring the mechanical and pharmacological drivers of restenosis [33, 34]. Across the corpus, authors frame their models as decision-support tools for personalized surveillance: patients flagged as high risk could be scheduled for earlier imaging follow-up or intensified lipid-lowering therapy, whereas low-risk individuals might avoid unnecessary repeat angiography. Although external validation is still rare, the methodological emphasis on class-imbalance handling and interpretable tree ensembles provides a reproducible template for future multicenter collaboration [35, 36].

#### Coronary artery identification

Coronary artery identification and lesion characterization was seen in 12 studies (21%). These investigations primarily leveraged imaging modalities (coronary angiography, CCTA, and chest x-ray sequences) to automate vessel segmentation, stenosis quantification, and occlusion detection. DL architectures such as convolutional neural networks, transformers, and hybrid models (e.g., UTNets, MI-MS ConvMixer, YOLO, U-Net) [15, 37] were employed to delineate coronary anatomy and highlight pathological regions. Performance metrics consistently demonstrated high accuracy, with Dice scores exceeding 0.90 in vessel segmentation tasks [38] and AUC values above 0.90 for stenosis detection, underscoring the clinical promise of these approaches. Feature inputs were dominated by pixel-level angiographic data, often enhanced with contrast agents or multi-frame sequences to improve vessel continuity and edge detection. Some models incorporated (QCA) as a reference standard, enabling comparative validation against established diagnostic benchmarks. Others explored chest x-ray–based risk scoring, extending identification beyond invasive imaging [39, 40].

### Research gap analysis

#### Limited sample size and single-center data

A dominant constraint in the review is the narrowness of patient cohorts. Thirty-two studies rely on relatively small or geographically restricted samples, often from a single hospital or research center [8, 20]. Researchers acknowledge that limited recruitment reduces statistical power, inflates confidence intervals, and makes subtle subgroup patterns invisible. More importantly, single-site data capture only the care pathways, imaging protocols, and demographic mix of that institution. When the same algorithm is applied in a setting with different scanners, treatment preferences, or disease prevalence, performance can drop sharply. Authors repeatedly call for multi-institution collaborations and federated learning approaches to enlarge datasets without breaching data-privacy regulations [13, 34]

#### Retrospective design and selection bias

Six studies highlight the inherent pitfalls of working with retrospective (EHR) extracts [19]. Because data are gathered after outcomes are known, confounding variables are harder to control and temporality can be ambiguous—was a medication started before or after symptom onset? Sampling frames can also be skewed toward patients who survived long enough or had comprehensive follow-up recorded. These biases may overestimate model accuracy and limit causal interpretation. Investigators recommend prospective registries or, when feasible, randomized controlled data collection to ensure consistent measurement and reduce hidden confounders.

#### Lack of external validation and generalization

Five studies admit they assessed their models only on internal splits [35]. Without testing on an entirely independent cohort, ideally from a different country, health-system, or technology platform, generalization remains speculative. The concern is that an algorithm captures idiosyncrasies such as institution-specific coding habits or imaging post-processing pipelines rather than disease biology. external validation ensures that predictive accuracy is not confined to a single institution or narrowly defined cohort, but instead reflects resilience across real-world heterogeneity. Transparent reporting of results from independent test cohorts should therefore be considered minimum standard in AI research. Authors therefore urge future work to include external test sets or model‐agnostic validation frameworks so that reported performance reflects real-world deployment conditions, as it strengthens confidence in model reliability, mitigates bias, and supports safe translation into clinical practice.

#### Incomplete indicators and missing data

Three studies report that important clinical, socioeconomic, or behavioral variables were either not recorded or contained excessive missing values [20, 34]. Missingness not only reduces sample size but can introduce bias if data are systematically absent in certain patient subgroups [10, 35]. Furthermore, algorithms trained on truncated feature spaces can over-rely on surrogate variables, leading to spurious associations. Suggested remedies range from improved EHR data-entry standards and linkage to public health databases, to imputation methods that explicitly model the missingness mechanism.

#### Challenges in integration and interpretability

A number of obstacles to clinical translation were identified in the 58 reviewed studies, especially in the areas of workflow integration, interpretability, and reimbursement. There was very little discussion of notification systems, clinician-facing interfaces, or (EHR) compatibility in the few studies that examined how their AI models could be integrated into real-time clinical workflows. Even highly effective models become less usable due to this lack of implementation planning. The usability of even high-performing models is compromised by this lack of implementation planning, with less than 10 studies reporting feature importance or used explainability techniques like SHAP (Shapely Additive Explanations) and LIME (local interpretable model-agnostic explanations), indicating interpretability as another significant gap. The majority used "black-box" architectures, such as CNNs and XGBoost (Extreme Gradient Boosting) [11, 15, 38]. without providing clinicians with information about how predictions were made. This undermines confidence and makes it more difficult to get regulatory approval. Along with the above concerns, no study discusses about the cost-effectiveness and payer engagements, they also do not document any pathway for obtaining approval of US Food and Drug Administration (FDA) and European Conformity (CE), without which these AI tools only remain only sound academically. Together, these obstacles show how important it is for future studies to focus on real-world deployment rather than just algorithmic performance. For AI in angiography to be not only accurate but also reliable, practical, and scalable in a variety of clinical settings, it will be crucial to integrate implementation science frameworks, interpretable modelling, and health economics.

#### Comparison with traditional risk scores

Only 5 of the 57 studies included in this scoping review explicitly compared artificial intelligence (AI) models with traditional clinical risk scores such as GRACE, SYNTAX, or TIMI [5, 6]. These comparisons were typically limited to retrospective, single-center designs and focused on outcome prediction following coronary angiography or percutaneous coronary intervention PCI. While AI models consistently demonstrated superior predictive accuracy, particularly in handling complex, multimodal datasets, the lack of standardized benchmarking and external validation limits the generalizability of these findings [20]. This highlights a critical gap in the literature: the need for robust, multicenter evaluations that directly assess the incremental value of AI over established scoring systems. Future research should prioritize head-to-head comparisons using harmonized datasets and prospective designs to determine whether AI can reliably augment or replace traditional risk stratification tools in diverse clinical settings.

#### Other methodological caveats

A final group of six studies lists assorted but noteworthy limitations: risk of overfitting when hyper-parameters are tuned on small development sets, insufficient model interpretability hindering clinician trust, class imbalance that forces resampling or cost‐sensitive learning, and the absence of cost–benefit or impact analyses in live clinical workflows [10, 20, 34]. While each study raises only one or two of these issues, collectively they underscore that technical validation alone is not enough; usability, fairness, and health‐economic evaluation are equally critical for safe translation to bedside practice.

## Discussion

**Fig. 2:**
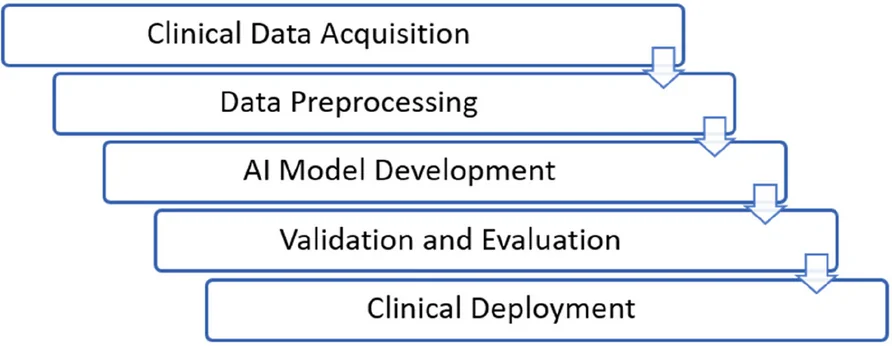
Conceptual artificial intelligence (AI) workflow

AI plays a versatile role in all areas of cardiology, as it enhances the utility of non-invasive diagnostics and reduces the need for expensive, invasive tests and procedures to diagnose CAD [41]. Doctors and other healthcare professionals can often experience burnouts due to high stress levels causing more medical errors, and high risk of medical errors, hence integration of automated tools as shown in Fig. 2 would help in reducing burnouts by reducing the workload on clinicians with respect to the time and quantity of reading as seen in [42]. AI can not only reduce burden on the clinicians with respect to time, but also has equal capability to predict or diagnose diseases as the doctors [43, 44]. Another major advantage of automated tools is the ability to analyze data in huge amounts; the amount of data being generated from the healthcare domain is huge and very complicated and analyzing such big volume of data can be difficult for humans.

In the field of cardiology, invasive coronary angiography is considered the gold standard diagnostic procedure for diagnosing CAD; this review aims to understand the trend of AI in predicting the outcomes of angiography procedures. Our study saw 18 studies with various models and patient data variables, which were beneficial and accurately predicted CAD. Apart from the automated tools hazard ratio and predictive scores are the statistical methods used to predict CAD or adverse events for treatment procedures.

One of the included studies compared the AI model with the GRACE score, showing the superiority of the AI model over the score in terms of long-term outcome prediction [6]. In agreement with the previous argument, [45] also surpasses the predictive value of PRAISE (Prediction of Adverse Events Followed by an Acute Coronary Syndrome) and GRACE 2.0. SYNTAX score is another method of assessment of CAD severity, helpful in determining the best possible revascularization method for the patient, use of AI model used have shown the capability to predict the SYNTAX score of the patient with good accuracy in the study by [46]. Across acute coronary syndrome, stable CAD, PCI follow-up, and high-risk subgroups such as chronic kidney disease, diabetes, AI demonstrates higher discriminatory power, better calibration, and more personalized risk estimation than GRACE, TIMI, or SYNTAX alone. The major advantage of AI is that it neutralizes any external sources of variance, such as human expert variability, and provides a consistent, standardized answers [47] thereby increasing the predictive power or efficiency of the method. AI also helps the cardiologists in segmentation and identification of missing coronary arteries [48, 49]. The predictive power of artificial models can help the cardiologist and the heart team to prepare for emergencies and possible complications during the procedure. The training of cardiologist in real life simulations can be facilitated by AI through a novel patient-specific technique for PCI procedures that makes advantage of similarity-based transfer learning [50].

 [51] developed and experimented with imputation method which improved the accuracy of the model by 19.8% for handling missing data and minimizing error, use of such data imputation methods can improve the accuracy and bring more confidence in use of AI in clinical settings. Improper data collection or omission of parameters can hugely impact the model behavior in different types of ethnicities, group of people or sex, leading to delivery of improper or no care to such patients [52]. Development and strict use and adherence to the standardized reporting formats available is crucial to address this gap [53].

The use of automate tools in clinical and healthcare settings comes with certain concerns with respect to data privacy, due to the sensitive nature of patient’s data, the data should be carefully stored and utilized for training and validation of various model. There still prevails a significant risk of reidentification of patients and data source in spite of data collected being masked or de-identified before sharing with third party data collector. Past studies have demonstrated the possibility of algorithm that could be used to reidentify 85.6% of adults and 69.8% of children in a physical activity cohort study, according to a 2018 study that examined data sets from the National Health and Nutrition Examination Survey, United States. This was true even though identifiers of protected health information were allegedly removed [54]. To prevent such incidences the laws for AI should be stringent and all encompassing, around the globe.

Majority of the studies in the review are retrospective, which introduces the possibility of selection bias. Various different types of bias in AI are already identified [47] and research is being carried to device new methods and techniques to minimize the bias [29]. Cohort selection bias is one of the main methodological problems and difficulties with the design of retrospective observational studies. This bias occurs when the study population is not chosen at random from the target population, when information is lost due to follow-ups, dropouts, or deaths, and/or when variables that could affect the outcome cannot be controlled. Another problem is the extent of the previously gathered data is another significant drawback of retrospective study designs. The majority of the data does not contain all pertinent information for analysis, which results in the omission of important confounders that may induce bias [30]. Data bias which is defined as any systematic and/or unjust difference in the way predictions are made for various patient populations that may result in differential care delivery [55] is another possible concern with the data set, which limits the capacity of the model to predict outcome, disease or mortality in a specific population of a group of population whose characteristics and not included or exposed to the models used in the study. Some studies even advise the readers to careful validation of such methods and suggest researchers to avoid over generalization and exercise caution in claiming cause-effect relationship in this kind of studies [56]. New comprehensive frameworks are being proposed to help outcome orientation of AI tools, use of which will be beneficial in better outcome ascertainment [57].

The scoping review highlights certain research gaps:

1. Limited sample size and single-center data prediction of outcomes: The studies included have conducted studies with small sample size and in single centers, signifying need for more studies with large sample sizes and multiple centers studies for better inclusion and accurate validation of models leading to better and reliable clinical application.
2. Selection bias in prediction of outcomes and missing data: The participants data collected in some studies is incomplete and the data collected also cannot adjust for the confounding variable and may introduce variations in application, requiring the need of robust standardized reporting format of the data being used for model building and validation.
3. Challenges in interpretability and integration: The studies in the review hardly discussed alerting systems, clinician-facing interfaces, or (EHR) interoperability.
4. Lack of comparison with conventional risk scores: Only few of the articles made a clear comparison between AI models and conventional clinical risk scores like GRACE, SYNTAX, or TIMI, leading to a question of validity.
5. Policy concerns: The algorithms need to be under stringent regulations for data security and privacy, so to keep the data safe and prevent sharing of data with third party agencies within the region and cross-country.
6. Publication trend: The study of publication trends region-wise shows the absence of publications from the African and South American countries and a major part of Asia, with only China contributing the majority of the publications; countries like India, wherein CAD is more common, should publish more.

This study has limitations including the absence of methodological and risk of bias evaluations and the resultant inappropriateness of the review to be used as evidence for clinical guidelines, limitations, and recommendations [9, 58, 59]. The scoping review also does not performing-depth analysis of the included studies. This study is of exploratory nature and does not include clinical guidelines.

## Conclusion

This scoping review emphasizes artificial intelligence's increasing capacity to accurately, consistently, and efficiently forecast the clinical outcomes of angiographic operations, providing important assistance in lowering clinical workload and decision fatigue. Small sample sizes, reliance on single-center data, selection bias, and ethical issues about data privacy are some of the drawbacks that are still apparent, and they all call for more confirmation through bigger, multicentric investigations, more randomized controlled trials and prospective data studies. Clinical applicability is further limited by issues with interpretability and workflow integration, such as the lack of clinician-facing interfaces, alerting systems, and EHR interoperability. Additionally, only a small number of studies have directly compared AI models with traditional risk scores like GRACE, SYNTAX, or TIMI, raising concerns about their practical value. Policy issues pertaining to privacy, data security, and regulatory oversight are still mostly unresolved, highlighting the necessity of strict frameworks to guarantee deployment that is both ethical and secure. Collectively, these gaps emphasize that while AI demonstrates promise in angiography, future research must focus on robust validation, transparent integration, and development and strong adherence to standardized reporting formats for minimizing data bias along with regulatory preparedness to enable reliable translation into clinical practice.

## Data Availability

No datasets were generated or analysed during the current study.

## Abbreviations

AI: Artificial Intelligence
AUC: Area under the curve
CAD: Coronary Artery Disease
CASP: Critical Appraisal Skills Programme.
CCTA: Coronary computed tomography angiography
CE: European Conformity
CNN: Convolution neural network
DAPT: Dual Anti-Platelet Therapy
DL: Deep Learning
EHR: Electronic Health Record
FDA: US Food and Drug Administration
GRACE: Global Registry of Acute Coronary Events
LIME: Local interpretable model-agnostic explanations
MACE: Major adverse cardiac events
MeSH: Medical Subject Headings
ML: Machine Learning
PCI: Percutaneous coronary interventions
PRISMA-Scr: Preferred Reporting Items for Systematic Reviews and Meta-Analyses Extension for Scoping Reviews
QCA: Quantitative coronary angiography
SHAP: Shapely Additive Expiations
SVM: Supportive vector machine
TIMI: Thrombolysis in myocardial infarction
XG: Boost Extreme Gradient Boosting
